# Deep transfer learning and data augmentation improve glucose levels prediction in type 2 diabetes patients

**DOI:** 10.1038/s41746-021-00480-x

**Published:** 2021-07-14

**Authors:** Yixiang Deng, Lu Lu, Laura Aponte, Angeliki M. Angelidi, Vera Novak, George Em Karniadakis, Christos S. Mantzoros

**Affiliations:** 1 School of Engineering, Brown University, Providence, RI 02912 USA; 2 Department of Chemical and Biomolecular Engineering, University of Pennsylvania, Philadelphia, PA 19104 USA; 3 Department of Medicine, Beth Israel Deaconess Medical Center, Harvard Medical School, Boston, MA 02215 USA; 4 Division of Applied Mathematics, Brown University, Providence, RI 02912 USA; 5 VA Boston Healthcare System, Harvard Medical School, Boston, MA 02215 USA

**Keywords:** Type 2 diabetes, Type 2 diabetes, Machine learning

## Abstract

Accurate prediction of blood glucose variations in type 2 diabetes (T2D) will facilitate better glycemic control and decrease the occurrence of hypoglycemic episodes as well as the morbidity and mortality associated with T2D, hence increasing the quality of life of patients. Owing to the complexity of the blood glucose dynamics, it is difficult to design accurate predictive models in every circumstance, i.e., hypo/normo/hyperglycemic events. We developed deep-learning methods to predict patient-specific blood glucose during various time horizons in the immediate future using patient-specific every 30-min long glucose measurements by the continuous glucose monitoring (CGM) to predict future glucose levels in 5 min to 1 h. In general, the major challenges to address are (1) the dataset of each patient is often too small to train a patient-specific deep-learning model, and (2) the dataset is usually highly imbalanced given that hypo- and hyperglycemic episodes are usually much less common than normoglycemia. We tackle these two challenges using transfer learning and data augmentation, respectively. We systematically examined three neural network architectures, different loss functions, four transfer-learning strategies, and four data augmentation techniques, including mixup and generative models. Taken together, utilizing these methodologies we achieved over 95% prediction accuracy and 90% sensitivity for a time period within the clinically useful 1 h prediction horizon that would allow a patient to react and correct either hypoglycemia and/or hyperglycemia. We have also demonstrated that the same network architecture and transfer-learning methods perform well for the type 1 diabetes OhioT1DM public dataset.

## Introduction

Type 2 diabetes (T2D) is a multifactorial progressive chronic metabolic disorder, accounting for approximately 90% of all cases of diabetes^1^. The prevalence of diabetes has been increasing rapidly over the past few decades^2^. In 2019, about 463 million adults were living with diabetes, while it is estimated to be 578 and 700 million by 2030 and 2045, respectively^3^. T2D and hyperglycemia are associated with an increased risk of vascular and non-vascular complications and premature mortality^4–6^. Furthermore, emerged evidence has also emphasized the importance of avoiding fluctuations in glycemia in T2D^7^. Of note, the Advanced Technologies & Treatments for Diabetes (ATTD) consensus recommendations highlight the role of glycemic variability and the time in ranges (including the time in target range, hyperglycemia, and hypoglycemia) as key metrics for Continuous Glucose Monitoring (CGM)^8^. The available antidiabetic treatments combined with a near-to-normal glucose levels approach, indicating the efforts of reducing high glucose levels and normalizing glycated hemoglobin levels in the absence of any contraindications, may lead to a lower frequency of T2D-related microvascular and macrovascular events^9,10^. On the other hand, intensified treatment targeting towards an intensive glucose control is associated with a higher risk of therapy-induced hypoglycemia and severe hypoglycemic events, which pose a potential risk for worsening or developing major macrovascular and microvascular complications, serious neurological consequences, as well as cardiovascular and all-cause mortality^11–14^. Additionally, hypoglycemia is a severe adverse outcome that may negatively impact a patient’s health and psychological status, leading to poor compliance and treatment adherence^13,14^. Hypoglycemic events are also associated with a high direct and indirect cost for patients, healthcare systems, and society^14,15^. Thus, the accurate prediction of blood glucose variations and, in particular, hypoglycemic events is of paramount importance to avoid potential detrimental complications and adjust the therapeutic strategy in a more optimized and personalized treatment strategy for patients with T2D. To this end, well developed predictive models with high sensitivity and accuracy, which are easy to implement, may facilitate better glycemic control, decrease the occurrence of hypoglycemic episodes or related complications and increase the quality of life in this population. Of note, due to the complexity of the blood glucose dynamics, the design of physiological models that produce an accurate prediction in every circumstance, i.e., hypo/normo/hyperglycemic events, is met with substantial restrictions.

Recently, machine learning has been shown to be very effective in solving classification and regression problems, and the ever-growing availability of already collected personal data makes the prediction of diabetic blood glucose through data-driven approaches possible^16–18^. Machine learning-based data-driven approaches use the individual’s recorded data, and require little understanding of the underlying physiological mechanism. Blood glucose dynamics in patients with type 2 diabetes are affected by factors such as pancreatic function, insulin levels, carbohydrate intake, history of dysglycemia and the level and extent of physical activity. Models using combinations of input parameters accounting for these factors have been previously considered^19,20^. Many different machine-learning methods have also been tested, including traditional machine-learning methods, e.g., auto-regression with exogenous input (ARX)^21^, support vector machines (SVM)^22^, Gaussian process (GP)^23^, and ensemble methods^24^, as well as deep-learning approaches, e.g., feed-forward neural networks (FNNs), recurrent neural networks (RNNs), and convolutional neural networks (CNNs). For more details of the studies until 2018, we refer the readers to relevant review papers^16–18^.

Owing to its predictive effectiveness, deep learning has quickly become quite effective in blood glucose prediction since 2018^19,21,25–30^. Among different deep-learning approaches, RNNs based on the long short-term memory (LSTM), have been designed for sequence prediction problems and are the most commonly used models^19,21,25,26,29^. However, there is no significant advantage observed by using the vanilla LSTM or convolution networks compared to a classic model (e.g., ARX), and in some cases RNNs or CNNs could showcase lower performance, as shown in a recent benchmarking study^21^. To achieve better prediction accuracy, more advanced network architectures have recently been developed, e.g., the recurrent convolutional neural network^27^, which includes a multi-layer CNN followed by a RNN, and GluNet^28^ based on the Wavenet architecture first presented in ref. ^31^.

Deep learning usually requires a large amount of data to train the networks, therefore, they are usually trained by population level rather than individual level data^18,27,28^. However, due to the variability of blood glucose dynamics among different patients and the heterogeneity of patient treatment response^32^, the models trained only by population level data cannot guarantee accurate prediction for each individual patient. To address the problem of *small dataset*, transfer learning^33–36^ can be employed, which stores knowledge gained while solving one problem (i.e., population data) and then applying it to a different but related problem (i.e., patient-specific data). Transfer learning has been employed in blood glucose prediction very recently^19,29,37–39^, but in these studies the patient-specific model based on transfer learning performed similarly to the population-based model or other classic machine learning models.

In addition to the problem of small data, another challenge in diabetic blood glucose prediction is the *data imbalance*. In particular, the dataset of normal-level blood glucose measurements (called *majority class*) is orders-of-magnitude larger than the dataset of blood glucose measurements with specific symptom (called *minority class*), e.g., hypoglycemia. The model trained on the imbalanced dataset leads to a biased performance, i.e., the accuracy of the minority class is much worse than that of the majority class^40^. To address the data imbalance issue, various general approaches have been developed^40–42^, including pre-processing approaches, algorithmic centered approaches, and hybrid approaches, but learning from imbalanced data effectively and efficiently is still an open problem^43^.

In this study, we tackle both the challenge of small datasets as well as the challenge of imbalanced datasets, by leveraging recent advances in deep learning and developing new methods for patient-specific prediction of diabetic blood glucose. First, we consider three neural network architectures and compare their performance systematically. These three representative architectures are RNNs with the GRU cell^44^, gated convolutional neural networks (CNNs)^45^, and self-attention networks (SANs)^46^, all of which show their unique advantages due to the difference in architecture designs for sequence classification, especially time-dependent sequences. Given the flexible structure of neural networks, we are presented with numerous ways of fine-tuning in the transfer-learning step. However, as noted in ref. ^47^, the performance of each fine-tuning technique is task-specific. To the best of our knowledge, there is no established consensus on the optimal fine-tuning technique for short-term glucose prediction. Hence, we develop four transfer-learning strategies for our glucose prediction task. Specifically, we examine the performance of these four transfer-learning techniques by comparing the results of predicting hypoglycemia vs. normoglycemia vs. hyperglycemia obtained from RNN, CNN, and SAN models in the setting of individual-based training. In addition, we consider new pre-processing approaches to address the data imbalance issue, because they are only performed on training data and can be directly combined with any neural network algorithm. Besides the common approach of re-sampling, where the training data is augmented by repeating existing samples, we also used other data augmentation techniques to generate synthetic data, including adding random noises and employing the recent techniques of mixup^48^ and time-series generative adversarial networks (TimeGAN)^49^. While mixup has been very popular in computer vision tasks^50^, TimeGAN is designed specially for time series prediction tasks. In this work, we test the performance of mixup and TimeGAN for data augmentation in the short-term blood glucose prediction task. To compare the performance of our algorithms with existing literature, we evaluate the proposed algorithms using a public dataset OhioT1DM^51^, documenting the CGM history and physiological measurements of 12 patients with type 1 diabetes. We also examine the performance of our algorithms on a private dataset recording blood glucose data for patients with type 2 diabetes. We include the details of our study design and blood glucose (BG) data collection in the Materials and Methods section. Taken together, herein we propose a model capable of predicting blood glucose variability in patients with type 2 diabetes with high sensitivity and specificity for the longest prediction horizon (time period after 30 min of BG collection) possible. More broadly, our combined methodology for tackling the fundamental problems of small and imbalanced datasets can be transferred to many other biomedical applications for predicting the outcomes of diseases using bio-signals and time-series data, e.g., classification of abnormal cardiac rhythms using data collected from wearable devices^52^ or electrocardiogram^53^, detection of seizure^54^ and Alzheimer’s disease^55^ using electroencephalography.

## Results

### Patient-specific prediction of blood glucose

**Fig. 1:**
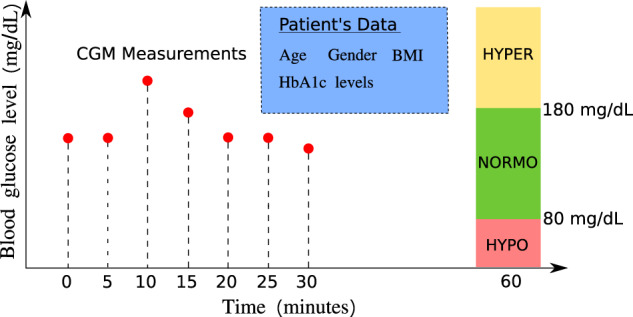
Patient-specific prediction of diabetic blood glucose. We use the patient’s blood glucose levels (every 5-min measurements) during several time periods in the past (e.g., 30 min) along with key and widely available patient’s personal data to predict the patient’s blood glucose level in the future (e.g., 30 min later). In particular, we aim to detect hyperglycemia (HYPER, blood glucose level > 180 mg/dL) and hypoglycemia (HYPO, blood glucose level < 80 mg/dL). NORMO, normoglycemia, 80 mg/dL ≤ blood glucose level ≤ 180 mg/dL.

According to Cox et al.^56^, severe hypoglycemia (SH) often follows a specific blood glucose fluctuation pattern that is identifiable from self monitoring blood glucose; hence, we consider the 30-min blood glucose as the primary predictor and the future glycemia as the target prediction. In this paper, we consider the following two classification tasks of diabetic blood glucose, i.e., one classification is “hypoglycemia” vs. “no hypoglycemia” and the other is “hypoglycemia” vs. “normoglycemia” vs. “hyperglycemia”, with the setup shown in Fig. 1. Specifically, the threshold for hyperglycemia is set to 180 mg/dL, i.e., blood glucose levels higher than 180 mg/dL are labeled with “hyperglycemia”. On the other hand, we set the threshold for hypoglycemia to be 80 mg/dL, i.e., we label blood glucose levels lower than 80 mg/dL as “hypoglycemia”. Here, unlike the common definition for level 1 hypoglycemia based on the threshold of 70 mg/dL, we instead choose 80 mg/dL as the hypoglycemia threshold. This is because recent results by Farrell et al.^57^ have revealed a measurement artifact, i.e., that the real-time continuous glucose monitoring (CGM), where we would expect these algorithms to have clinical applicability, underestimates the degree of hypoglycemia by a difference of 10 mg/dL, as shown in Supplementary Fig. 1.

### Deep transfer learning for small patient-specific data

**Fig. 2:**
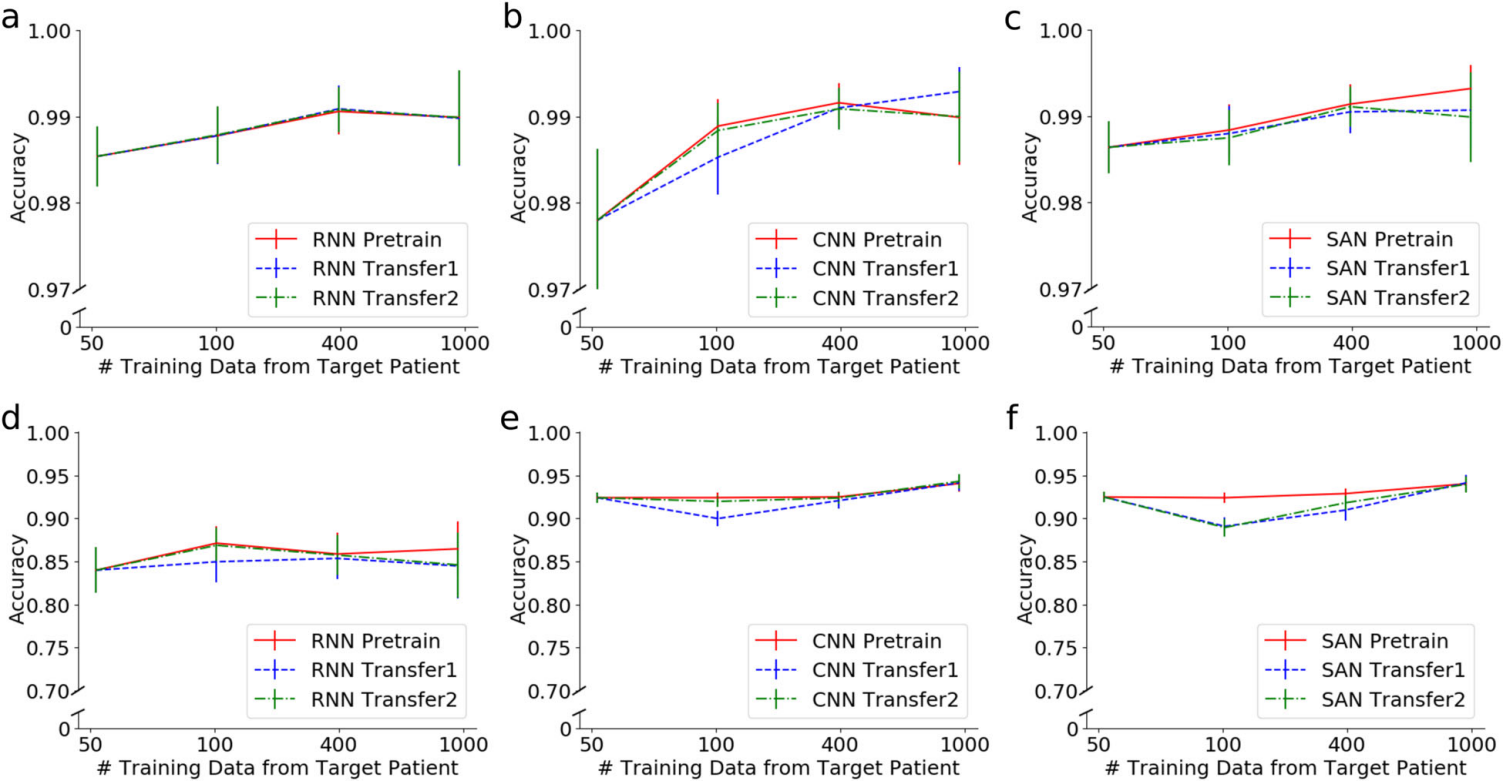
Prediction accuracy comparison among different architectures (RNN, CNN, and SAN) with respect to the number of training data from the target patient in two classification tasks. **a** to **c** Prediction accuracy of the binary classification, i.e., identifying the neural network output as hypoglycemia or no hypoglycemia, using **a** RNN, **b** CNN, and **c** SAN. **d** to **f** Prediction accuracy of the three-class classification, i.e., identifying the neural network output as hypoglycemia or normoglycemia or hyperglycemia, using **d** RNN, **e** CNN, and **f** SAN. The data from a target patient is divided into two parts, one is for training and the other is for testing, and the prediction horizon is fixed at 30 min. The accuracy of Transfer3 is lower compared to other transfer-learning methods. Here, CNN Pretrain and Transfer2 as well as SAN Pretrain show the best performance. Error bars (standard deviation, s.d.) are computed over all patients’ results.

We compare the performance of three neural network architectures by the averaged prediction accuracy per capita for these two classification problems. The results in Fig. 2 suggest that as the training data size from the target patient increases, the prediction accuracy of all models generally increases. We note that CNN models are generally more accurate than RNN models and slightly outperform SAN models with higher mean and smaller standard deviation for prediction accuracy in both of the classification tasks. The results also suggest that the transfer-learning models (Transfer1 and Transfer2) can sometimes outperform the pre-trained models in CNN models. We also compared our models with some existing classification methods, i.e., logistic regression, GP, SVM, and FNN in terms of (1) Predicting hypoglycemia vs. no hypoglycemia (Supplementary Table 1); (2) Predicting hypoglycemia vs. normoglycemia vs. hyperglycemia (Supplementary Table 2) over a prediction horizon of 30 min; (3) Predicting hypoglycemia vs. no hypoglycemia (Supplementary Table 3); (4) Predicting hypoglycemia vs. normoglycemia vs. hyperglycemia (Supplementary Table 4) over a prediction horizon of 60 min. In both tasks, our models showed consistent increases in accuracy and the area under the receiver operating characteristic curve (AUROC) given more training data from the target patient and, specifically, better than those by existing classification methods examined in predicting hypoglycemia vs. normoglycemia vs. hyperglycemia, see Supplementary Tables 1 and 2.

**Fig. 3:**
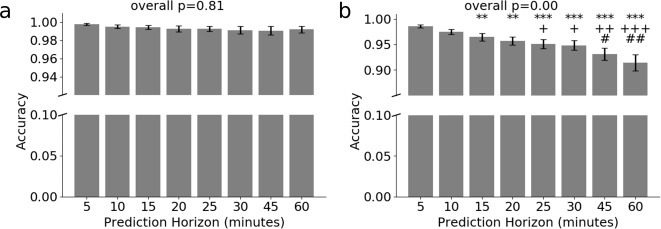
Prediction accuracy for two classification tasks given different prediction horizons using the best CNN model. Prediction accuracy for **a** binary classification, i.e., identifying the neural network output as hypoglycemia or no hypoglycemia, and **b** three-class classification, i.e., identifying the neural network output as hypoglycemia or normoglycemia or hypoglycemia, given different prediction horizons. No statistical significance is observed for binary classification. *p-value ≤ 0.05; **p-value ≤ 0.01; ***p-value ≤ 0.001, in comparison to the prediction horizon at 5 min; ^+^*p*-value ≤ 0.05; ^++^*p*-value ≤ 0.01; ^+++^*p*-value ≤ 0.001, in comparison to the prediction horizon at 10 min and ^#^*p*-value ≤ 0.05; *##p*-value ≤ 0.01; ^###^*p*-value ≤ 0.001, in comparison to the prediction horizon at 15 min. Error bars (standard deviation, s.d.) are computed over all patients’ results.

**Fig. 4:**
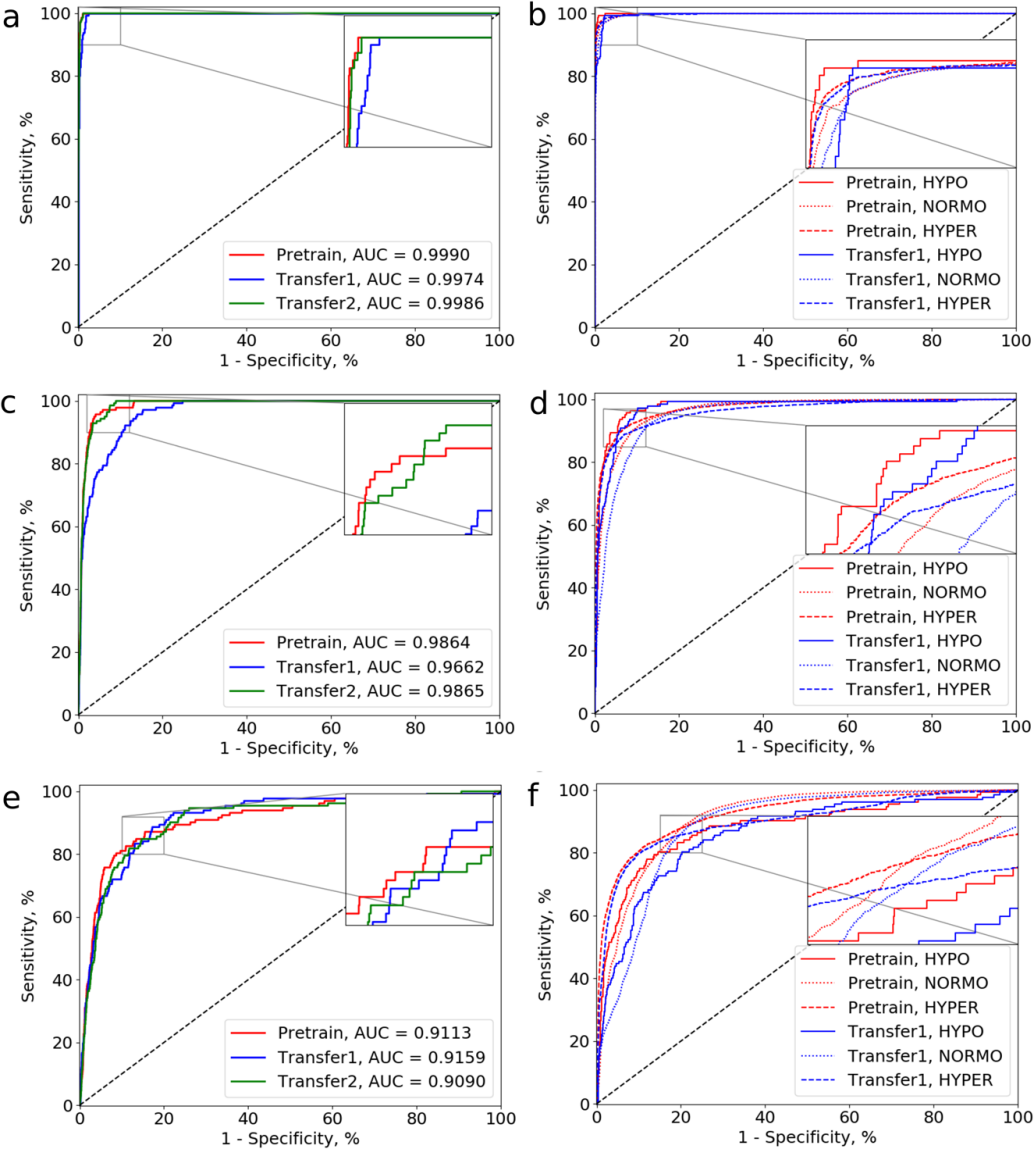
ROC curves for two classification tasks given prediction horizons at 5, 30, and 60 min using the best CNN model. **a**, **b** Examples of the ROC curves for the prediction horizon at 5 min, in **a** binary classification and **b** three-class classification. **c**, **d** Examples of the ROC curves for the prediction horizon at 30 min, in **c** binary classification and **d** three-class classification. **e**, **f** Examples of the ROC curves for the prediction horizon at 60 min, in **e** binary classification and **f** three-class classification. AUC, area under the ROC curve. Binary classification denotes predicting hypoglycemia vs. no hypoglycemia. In three-class classification, we iterated over the labels (HYPO for “hypoglycemia”, NORMO for “normoglycemia” and HYPER for “hyperglycemia”) to compute the ROC curves. The results by Transfer2 is comparable to those by Pretrain while those by Transfer3 are worse than Transfer1 and Transfer2, hence we only show the results of Pretrain and Transfer1 for brevity.

Figure 3 shows the sensitivity analysis of the prediction horizon on the prediction accuracy and Fig. 4 shows the ROC curves (receiver operating characteristic curves) of best models among all the models tested, given the training data size from the target patient around 1000 data segments. Figure 3a suggests that the sensitivity between different prediction horizons is negligible in predicting hypoglycemia vs. no hypoglycemia (binary classification), while Fig. 3b shows that the sensitivity between different prediction horizons becomes larger when the time elapse of two prediction horizons is large in predicting hypoglycemia vs. normoglycemia vs. hyperglycemia (three-class classification). Figure 4 suggests that our best model maintains a high AUROC in both classification tasks for a range of clinically useful prediction horizons, i.e., 5 min (Fig. 4a, b), 30 min (Fig. 4c, d) and 60 min (Fig. 4e, f).

We evaluate the performance of our models on the OhioT1DM dataset, a de-identified public dataset recording the glucose level, insulin dosage, exercise and other metabolism readings for six patients with type 1 diabetes in the 2018 version and another six patients with type 1 diabetes in 2020 version^51^. We demonstrate the performance of our model by evaluating it on the dataset of six patients in the 2020 version. Specifically, the training data is the union of all the training data of the 12 patients and the testing data of the 6 patients in the 2018 version. We discard any training sequences with one or more missing data points. The training process of our model is again two-step, i.e., in the first training step, we pretrain the model on the training data excluding the training data of the patient to be tested; in the second training step, we fine tune the model on the training data of the patient to be tested; finally we test the model on the testing data of the target patient. To make a fair comparison, we compare our model performance with those using historical blood glucose levels as the only predictor and 30 min as the sampling horizon^58–61^. Similarly, all models are evaluated on 6 patients in the 2020 version, see Table 1.

**Table 1:** Performance comparison between our best model and other models using only blood glucose levels as the model input on OhioT1DM dataset.

(a): Regression results for prediction horizon at 30 and 60 min.					
Metrics	Ours	Bevan et al.^59^	Khadem et al.^58^	Joedicke et al.^60^	Ma et al.^61^
30 min MAE	13.53	14.37	14.14	15.50	14.52
30 min RMSE	19.08	18.23 (18.82^a^)	19.40	24.51	20.03
60 min MAE	24.65	25.75	25.32	24.78	26.40
60 min RMSE	33.80	31.10	33.91	38.66	34.89

(b): Binary classification results for prediction horizon at 30 min.		
Metrics	Ours	Bevan et al.^59^
Accuracy	95.98%	95.65%
F1 score	61.72%	57.40%
Sensitivity	59.19%	49.94%
Precision (PPV)	67.68%	69.00%
Specificity	98.15%	98.61%
NPV	97.55%	96.76%

*MAE* mean absolute error, *RMSE* root mean squared error, *PPV* positive predictive value, *NPV* negative predictive value.

^a^Denotes the 30 min RMSE of the model by Bevan et al.^59^ without imputing missing values with the mean of training dataset.

(**a**) Our model outperforms other models in terms of mean absolute error in both 30 and 60 min prediction horizon. We report the mean of the results over five different runs. (**b**) Our model for binary classification (hypoglycemia vs. no hypoglycemia) outperforms that by Beven et al. in terms of accuracy, sensitivity, and F1 score of the positive class (the hypoglycemia class). The classification results are obtained by setting a 80 mg/dL threshold to the blood glucose level, i.e., blood glucose level 80 mg/dL with negative class (no hypoglycemia class). Our best model is CNN + Transfer2.

The results in Table 1a suggest that our best model (CNN + Transfer2) outperforms all other models in terms of mean absolute error (MAE) in both cases of 30 and 60 min prediction horizon. While the root mean squared error (RMSE) of our results are not the best among these five models, they are the second to the best model, i.e., the model by Bevan et al.^59^, which reported RMSE of 18.23 for 30 min prediction horizon after imputing missing value with mean value of the training dataset. Bevan et al. also reported a slightly higher RMSE at 18.82 for 30 min prediction horizon when missing data in the training sequence is discarded, which we believe is very close to our results, given the same missing data handling strategy. It is standard practice in clinical medicine to use sensitivity to evaluate the value of a test as a screening test and use specificity to evaluate the value of a test as a confirmatory test. Hence, we further examine our model performance using the regression results for binary classification of hypoglycemia class vs. no hypoglycemia class. Specifically, we set the threshold of hypoglycemia vs. no hypoglycemia to be 80 mg/dL, i.e., values < 80 mg/dL are denoted as the positive class while greater than that as the negative class. The results of the binary classification for 30 min prediction horizon in Table 1b suggest that our model is more accurate than that by Bevan et al., i.e., higher accuracy and F1 score. Compared to the results of Bevan et al., our model shows same specificity and negative predictive value (NPV) but much better sensitivity (almost 10% higher). Hence, our model provides a better screening test (sensitivity) and equally good confirmatory test (specificity), which is an overall better test and is highly favorable in the clinical setting.

### Improvement of sensitivity for imbalanced data

In this section, we show further detailed analysis with regression-based models for classification, i.e., we perform regression prediction then convert the real-valued prediction into class labels, as shown in Fig. 1. We note that our raw BG data is innately real-valued, hence it is natural to investigate the data feature following a regression approach. Here, we aim to show the effects of different data augmentation methods mainly on the minority dataset. With our previous classification analysis, we set up the regression model with the following preconditions: the prediction horizon is 20 min if not mentioned otherwise and the hypoglycemia threshold is set to be 80 mg/dL. We will show results without transfer learning, i.e., we train the models on the dataset, which is the union of other patients’ data except for the target patient and then directly test on the target patient’s dataset. We focus on comparing the model performance in predicting hypoglycemia vs. no hypoglycemia by converting the real-valued prediction into two labels: one label is “hypoglycemia”, meaning the prediction is below 80 mg/dL while the other is “no hypoglycemia”, meaning the prediction is above or equal to 80 mg/dL. We also carry out the same conversion on the true BG values measured by the CGM. With the conversion, we can then compare four classification scores, sensitivity, positive predictive value, specificity, and negative predictive value between different models.

#### Selection of loss functions

**Fig. 5:**
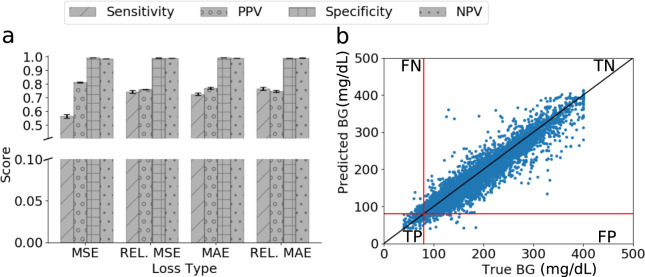
Regression performance of the best CNN model on the original dataset (no augmentation on the training dataset), using four different loss functions. **a** Performance comparison between four loss functions, i.e., mean squared error (MSE), relative mean squared error (REL. MSE), mean absolute error (MAE) and relative mean absolute error (REL. MAE) in terms of four prediction scores, i.e., sensitivity, positive predictive value (PPV), specificity and negative predictive value (NPV). The results suggest that the relative mean absolute error (REL. MAE) serves as the best loss function in that it maintains good and balanced performance regarding the four scores shown. Hence, we keep the REL. MAE as the loss function for the subsequent analysis. Error bars (standard deviation, s.d.) are computed over all patients’ results. **b** True blood glucose values measured by CGM vs. the predicted blood glucose values using REL. MAE as the loss function. The blue scatter points denotes the measurement-prediction pairs. The black diagonal line denotes the perfect prediction. The red lines denote the hypoglycemia threshold 80 mg/dL.

We tested the performance of four different loss functions, i.e., mean absolute error, relative mean absolute error, mean squared error and relative mean squared error using the original training dataset without data augmentation. In particular, we examined the performance of models with different loss functions using four classification metrics, i.e., sensitivity, positive predictive value (PPV), specificity and negative predictive value (NPV). To compute these four classification metrics, the real-valued blood glucose prediction is categorized into two classes, i.e., “hypoglycemia” (positive class) and “no hypoglycemia” (negative class). Figure 5a shows the comparison of model performance using different loss functions. The result suggests that the model using *relative mean absolute error* (REL. MAE) outperforms models using the other three loss functions, because the model using the relative mean absolute error maintains a balanced high value for each of the aforementioned four metrics. Figure 5b shows the scatter plot of true BG vs. predicted BG also suggests high prediction accuracy with the points clustering near the diagonal black line indicating the perfect prediction. The red lines divide the whole domain into four rectangular regions, i.e., the true positive region (TP) denoting that the true BG is “hypoglycemia” and prediction is also “hypoglycemia”; the false-positive region (FP) denoting that the true BG is “no hypoglycemia” but the prediction is “hypoglycemia”; the false-negative region (FN) denoting that the true BG is “hypoglycemia” but the prediction is “no hypoglycemia”; the true-negative region (TN) denoting that the true BG is “no hypoglycemia” and the prediction is “no hypoglycemia”.

#### Data augmentation

In this part, we fix the loss function in our model to be the *relative mean absolute error* (REL. MAE) and compare the performance of our model when four different data pre-processing techniques are implemented for data augmentation on the training data of the minority class and a prediction horizon at 20 min.

**Fig. 6:**
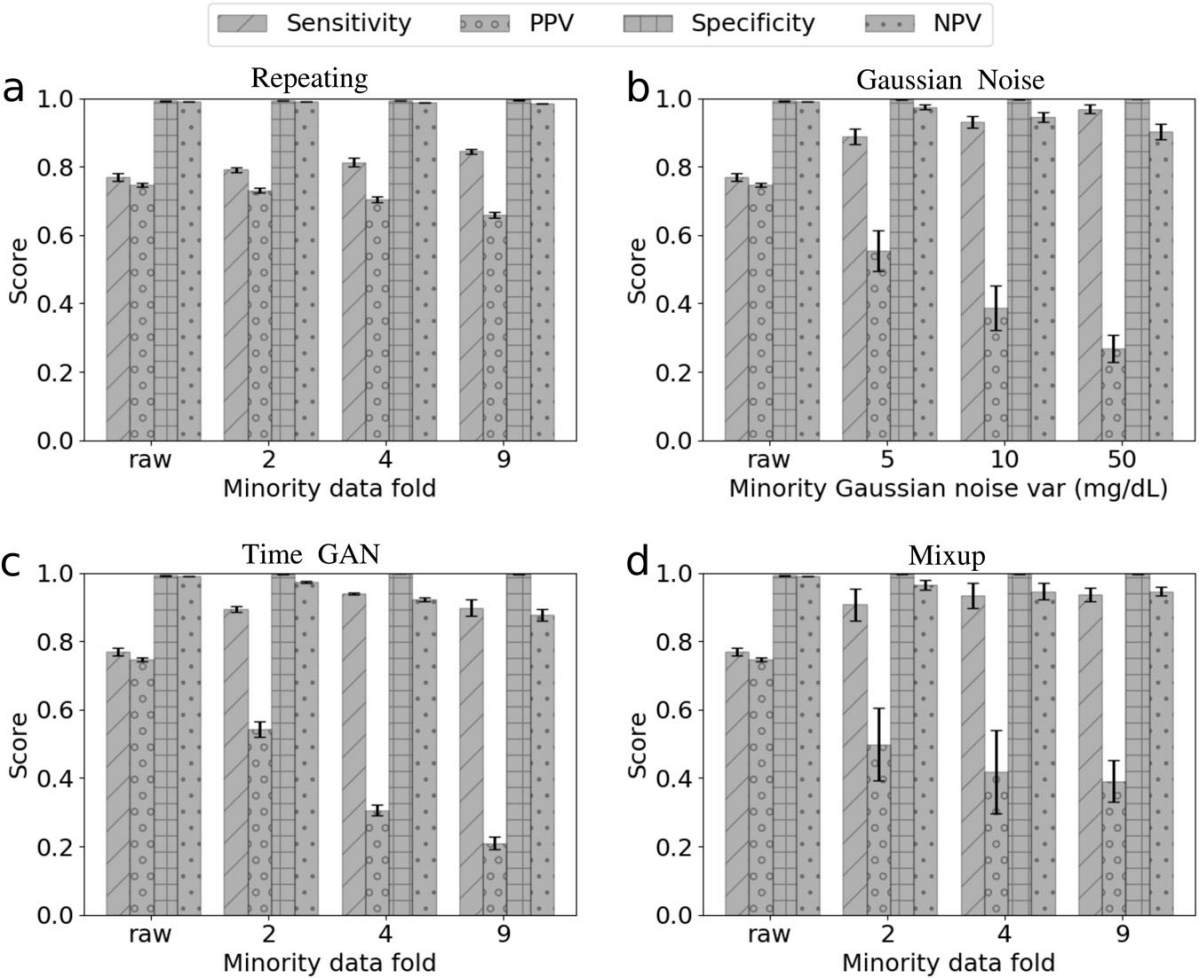
Predictive scores by different data augmentation methods and different folds of minority data augmentation. Hypoglycemia (the minority class, also the positive class) samples in the training data is augmented with **a** oversampling by repeating, **b** Gaussian noise infusion, i.e., the size of the minority training data is doubled by adding a copy of the raw minority data contaminated by Gaussian noises of different levels, **c** TimeGAN, and **d** mixup (*α* = 2), while “no hypoglycemia” (majority class, also the negative class) samples remain intact. The minority data fold represents the number of copies of hypoglycemia samples in the training data after data augmentation. “Raw” denotes no data augmentation on the training dataset; twofold denotes that the raw minority data is kept in the training data and another copy of minority data is generated by either repeating or synthesizing in each training epoch. The mean and standard deviation of the classification metrics are obtained with five different runs. A table recording the detailed numerical results can be found in Supplementary Table 5. Error bars (standard deviation, s.d.) are computed over all patients’ results.

For this data augmentation method, we repeat the minority samples (the input-output pairs where the output BG is less than 80 mg/dL) in the training dataset for *k* folds, i.e., for two-fold oversampling by repeating, we duplicate the minority samples once such that the minority data is doubled in the augmented training dataset. Hence, for *k*-fold oversampling by repeating, we augment the training data by adding *k* − 1 copies of the training data labeled as hypoglycemia (output BG < 80 mg/dL) to the augmented training dataset. Figure 6a shows that oversampling by repeating only improved slightly in the sensitivity when the minority augmentation fold increases, which is different from the other three augmentation methods.

Adding Gaussian white noises to the training dataset has been proved to be an effective way of data augmentation for CNNs^27^, and specifically for CNNs using wearable sensor data^62^. In this part, we tried different levels of Gaussian white noises distinguished by the variance of the noise. In particular, we infused white noises with variance at 5, 10, 50 mg/dL, respectively, to the input BG data of minority class, whose output BG value is below the hypoglycemia threshold, i.e., there are two copies of minority training data in the augmented dataset, one is the original copy collected by the CGMs, and the other is a copy generated by infusing Gaussian noises. Figure 6b suggests that increasing the variance of the infused Gaussian noise will increase the sensitivity of the model.

We generated synthetic minority samples using TimeGAN^49^, by training a TimeGAN using the original minority samples in our dataset. TimeGAN combines the versatility of the unsupervised GAN approach with the control over conditional temporal dynamics afforded by supervised auto-regressive models, by leveraging the contributions of the supervised loss and jointly trained embedding network, and hence can generate realistic time-series data. Our trained TimeGAN is validated by the PCA and T-NSE plots for the original minority samples and synthetic minority samples, see Supplementary Fig. 3. We then compared the performance of models when different folds of synthetic minority samples were added to augmented training dataset. Figure 6c shows that adding more minority data generated by TimeGAN could also improve model sensitivity but not as monotonically as the other methods tested.

Zhang et al.^48^ recently introduced mixup to improve the generalization of neural network architectures, by linearly interpolating between samples in the training dataset using the following formula,

1 $$\tilde{x}=\lambda \ {x}_{i}+(1-\lambda ){x}_{j},\ \tilde{y}=\lambda \ {y}_{i}+(1-\lambda ){y}_{j},$$

where $\tilde{x},\tilde{y}$ denote generated input and output, respectively; *λ* is a hyperparameter following the Beta distribution, Beta(*α*, *α*); *x*_*i*_, *x*_*j*_ denote inputs from two different samples and *y*_*i*_, *y*_*j*_ denote the corresponding output of those two different samples. We note that in the original mixup algorithm, *y*_*i*_, *y*_*j*_ can be of different class, while in our model we only perform mixup on the minority class, i.e., *y*_*i*_, *y*_*j*_ satisfy the condition that *y*_*i*_ < 80 and *y*_*j*_ < 80.

There have been some attempts to perform data augmentation using mixup in time-series analysis of biosignals, such as electroencephalogram (EEG) and electrocardiogram (ECG)^63^, generating virtual biosignals from real biosignals of different types^64^. While in this work, we implement mixup for data augmentation on minority class only to alleviate the effect of data imbalance. By *k*-fold mixup, the size of the minority class is increased to *k* times of its original size by adding *k* − 1 copies of synthetic data using mixup for each training epoch. The original mixup algorithm does not include *k* as a hyperparameter, i.e., in the original mixup, the original training data is replaced by synthetic data generated by linear interpolation in the beginning of each training epoch. Figure 6d shows that increasing the folds of minority data by mixup could help improve model sensitivity but the uncertainty in the positive predictive value is relatively larger than other augmentation methods.

**Fig. 7:**
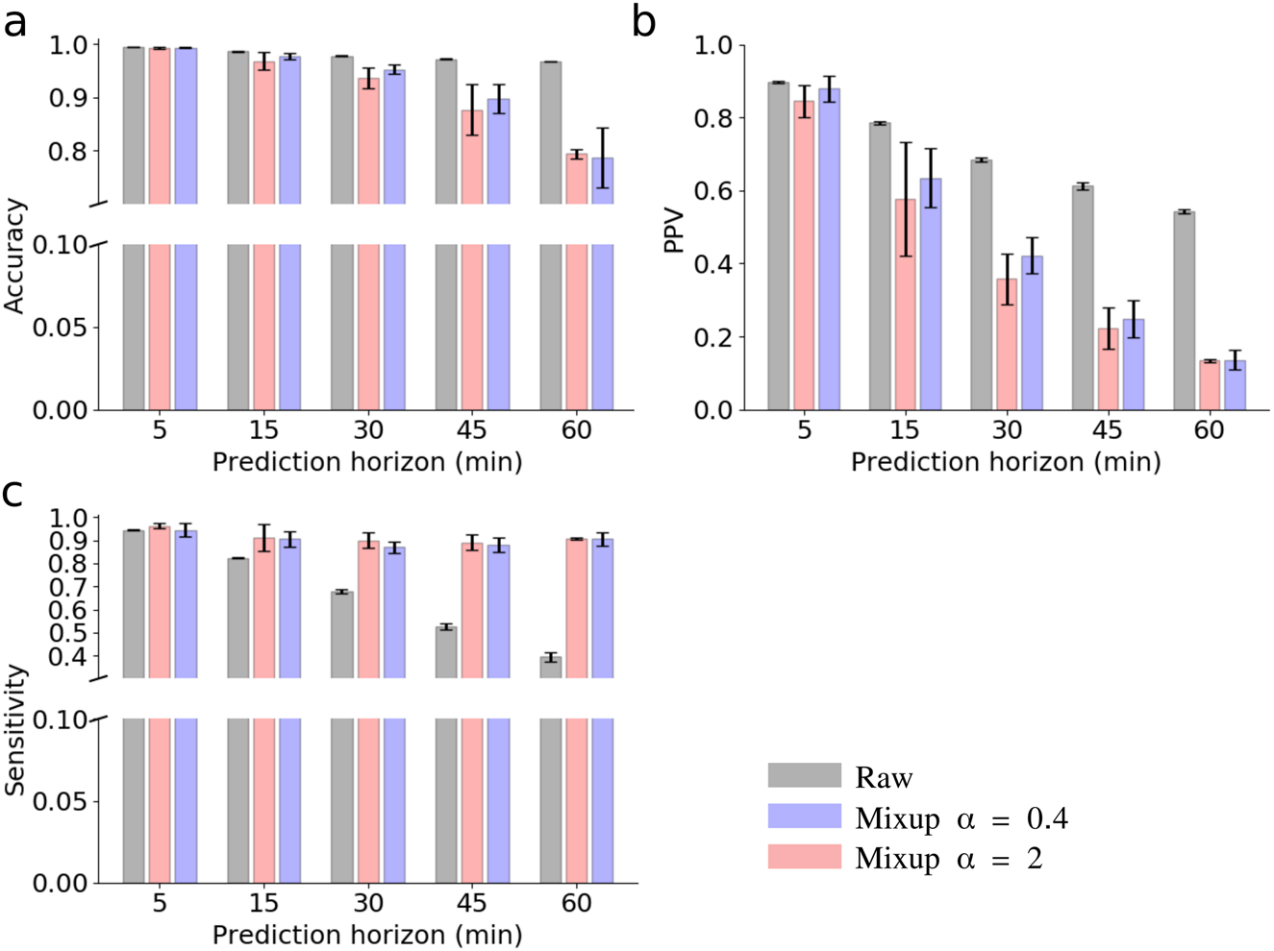
Sensitivity analysis of the prediction horizon on three predictive scores with twofold mixup data augmentation on minority training data. We compare the performance of our CNN model trained on the raw training dataset and on the training dataset augmented by two mixup models, one with *α* = 0.4 and the other with *α* = 2 for the Beta distribution Beta(*α*, *α*) implemented in mixup. The performance of each model is calibrated in terms of **a** prediction accuracy, **b** positive predictive value (PPV, the precision of the positive class), and **c** sensitivity (recall of the positive class). A table for the detailed numerical results is shown in Supplementary Table 6. Hypoglycemia (the minority class, also the positive class) samples in the training data is augmented with twofold mixup. The purple-shaded bars denote the predictive scores by mixup (*α* = 0.4), the red-shaded bars denote those by mixup (*α* = 2), and the gray-shaded bars denote those by the raw training data (no data augmentation). Error bars (standard deviation, s.d.) are computed over all patients’ results.

The hyper-parameter *α* in the Beta distribution Beta(*α*, *α*) of mixup is a very sensitive parameter controlling the diversity of the synthetic samples, i.e., higher *α* produces samples more resembling to the reference real data while lower *α* introduces samples very different from the reference real data. With *α* = 1, Beta(1, 1) is equivalent to a uniform random distribution. Here, we compare the performance of our model given *α* = 0.4 and *α* = 2 in twofold mixup, in terms of two classification scores, i.e., positive predictive value (PPV) and sensitivity for the positive class (the minority class, hypoglycemia samples), and examine the sensitivity of those two classification scores for different prediction horizons. The results for *α* = 0.4 and *α* = 2 are shown in Fig. 7. We note that mixup with either *α* = 0.4 or *α* = 2 improves the model sensitivity over different prediction horizons. Specifically, models trained on the training dataset augmented by mixup show high sensitivity within all the prediction horizons examined while the model without data pre-processing shows decreased sensitivity over longer prediction horizons. The model trained on the training dataset augmented by mixup *α* = 0.4 shows different uncertainty in the predictive scores for different prediction horizons; for example, the standard deviation of sensitivity and PPV for prediction horizon at 15 min are much larger than those for other prediction horizons. However, the model trained on the training dataset augmented by mixup *α* = 2 shows similar uncertainty in the predictive scores among different prediction horizons, mainly because samples generated by mixup *α* = 0.4 are relatively distinct from the original samples collected while those by mixup *α* = 2 is similar to the original samples, hence preserves the data patterns. As the prediction horizon increases, the sensitivity of the model decreases while the PPV increases when training on the raw dataset. However, the models trained on datasets augmented by mixup show high sensitivity and a gradual drop in the PPV, regardless of the increase in the prediction horizon.

The results in Fig. 6 indicate that by adding more training data of minority class, either through duplication or synthesizing, will increase the model sensitivity but decrease the positive predictive value, i.e., the precision for minority class. Specifically, given the same amount of minority samples in the training data, the increase in model sensitivity and decrease in precision for minority class is more significant in those with synthetic minority samples, compared to the oversampling by repeating. These results prove a recent finding that transforms (augmentations), which preserve the labels of the data can improve estimation by enlarging the span of the training data^50^. In our case, we preserve the labels of the data by only augmenting the minority training data, which consequently increases the span of minority data, by generating synthetic data using Gaussian noise, TimeGAN or mixup. Our results also suggest that synthetic minority data (data generated by infusing Gaussian noise, TimeGAN or mixup) could increase the span of minority data much more significantly than repeating the original minority data.

## Discussion

Type 2 diabetes is considered an epidemic worldwide. Hyperglycemia selectively damages cells that are not able to reduce glucose transport into the cell, such as capillary endothelial cells in the retina, mesangial cells in the renal glomerulus, and neurons and Schwann cells in peripheral nerves. High intracellular glucose concentration leads to the exhaustion of the antioxidant pathways, altered regulation of gene transcription and increased expression of pro-inflammatory molecules resulting in cellular dysfunction and death^65^. On a clinical level, these cellular changes translate into micro and macrovascular complications of diabetes associated with poor outcomes and increased mortality^66^. Current diabetes treatment regimens may decrease the occurrence of complications associated with hyperglycemia, however, they also suppose a risk of extremely low glucose levels. Hypoglycemia can lead to permanent neurological damages if not treated promptly and increased mortality^13^. The prediction of blood glucose variations helps to adjust acute therapeutic measures and food intake in patients with type 2 diabetes.

We developed transfer-learning methods to predict “hypoglycemia” vs. “no hypoglycemia” or “hypoglycemia” vs. “normoglycemia” vs. “hyperglycemia” for patients with type 2 diabetes. We obtained state-of-the-art results by tackling two major challenges associated with the small data size for individual patients as well as the imbalanced datasets, i.e., small samples for hypoglycemia. To deal with small datasets, we considered three neural network models, including recurrent neural networks (RNNs), convolutional neural networks (CNNs) and self-attention networks (SANs). We also examined four transfer-learning strategies, which enabled us to train the neural networks with a small amount of individual’s recorded data. We demonstrated the performance of our methods on the data obtained from 40 patients. We achieved high prediction accuracy for the task of predicting hypoglycemia vs. no hypoglycemia with accuracy no less than 98% and AUROC  greater than 0.9 for all the prediction horizons examined. For the task of predicting hypoglycemia vs. normoglycemia vs. hyperglycemia, the best model among all tested models achieved high accuracy greater than 89% and AUROC greater than 0.86, for all the prediction horizons examined (up to 1 h). Our results suggest that as the prediction horizon prolongs, the prediction accuracy, as well as the AUROC decreases, as expected, in both classification tasks.

When comparing the model performance on predicting hypoglycemia vs. no hypoglycemia and predicting hypoglycemia vs. normoglycemia vs. hyperglycemia, our results suggest that the overall prediction accuracy and AUROC in the task of predicting hypoglycemia vs. no hypoglycemia is always higher than those in the task of predicting hypoglycemia vs. normoglycemia vs. hyperglycemia.

More specifically, statistical significance was observed between two short prediction horizons (5 and 10 min) and the largest prediction horizon (60 min) in the task of predicting hypoglycemia vs. normoglycemia vs. hyperglycemia. We note that despite of the statistical differences observed among different prediction horizons, the model always maintained high accuracy.

However, a closer examination on our dataset reveals that most of the blood glucose levels are labeled as either normoglycemia or hyperglycemia and hence only very few blood glucose levels are labeled as hypoglycemia, making hypoglycemia the definite minority class, resulting in models with sensitivity around 77% and positive predictive value around 75% for a prediction horizon at 20 min. Given the need to detect hypoglycemia more accurately and robustly, data augmentation on the minority class, i.e., augment the hypoglycemia samples in our training dataset, is an effective way of enforcing the neural networks to learn the underlying patterns of the hypoglycemia data at a finer scale compared to learning on the dataset without data augmentation. Our tests suggest that data augmentation on the minority class using synthetic data (not oversampling by repeating) increases the model sensitivity in detecting hypoglycemia, from more than 80% to less than 96% depending on the specific augmentation method for a prediction horizon at 20 min. This allows early treatment intervention and prevention of potential hypoglycemic events and hence is a significant improvement preferred in clinical diagnosis given the fatal consequences of hypoglycemia for patients with serious complications caused by type 2 diabetes. However, given the imbalance nature of our dataset, the increased sensitivity, i.e., the recall of the minority class, observed from models trained on the augmented dataset also comes with a decrease in the positive predictive value, i.e., the precision of the minority class. Although the trade-off between the precision and recall for imbalanced datasets is a commonly observed dilemma, with minority data augmentation of different folds, we could still achieve a good balance between those two metrics such that they are acceptable in practical scenarios.

Despite the high accuracy and a few training data demanded by our method, there are some limitations to current work. Different from other physiologically derived approaches, this method is purely data-driven with no physiological knowledge, and performs prediction merely based on the blood glucose history. It is recognized that data-driven methods are double-edged swords. On one side, data-driven methods relieve physicians from exhausting all possible combinations of physiological inputs given large samples or data. On the other side, it is not an easy task to incorporate domain knowledge to data-driven methods, especially in neural network-based models. In our study, we identify nutritional intake, exercise or stress conditions in dysglycemia prediction as the domain knowledge, the appropriate incorporation of which could possibly improve the model accuracy. Hence, we will propose the development of physiologics-informed neural network models in our future work. This and similar methods in the future are expected to have important clinical implications in terms of preventing and avoiding this potentially lethal complication, e.g., through alerts generated directly to the patient or by linking the prediction algorithms to the programmable insulin pumps.

To summarize, we proposed a new method for predicting hypoglycemia vs. no hypoglycemia and predicting hypoglycemia vs. normoglycemia vs. hyperglycemia, and the method shows remarkable performance characterized by high prediction accuracy and AUROC as well as other metrics, including specificity and sensitivity. In particular, a combined approach of transfer learning and data augmentation for imbalanced data can be proved a very powerful new framework for short term predictions for type 2 diabetes. Here, we focused on time periods up to 60 min, with a notable sensitivity and positive predictive value of the model observed during the first 15 and 30 min. We believe that accurate hypoglycemia prediction over this period of time offers the most in terms of having potential warning signs and preventing adverse events by hypoglycemia. By incorporating transfer learning, this method could provide patient-specific results in both predicting hypoglycemia vs. no hypoglycemia and predicting hypoglycemia vs. normoglycemia vs. hyperglycemia with relatively few patient-specific training blood glucose samples. For example, in our case, we used 1000 time segments, equivalently 83 h long, from the target patient.

## Methods

### Dataset

The use of blood glucose (BG) history of patients with T2D in this study were approved by the institutional review board (IRB) of the Beth Israel Deaconess Medical Center. Informed consents were obtained from all human participants. The BG level was measured every 5 min by a Continuous Glucose Monitoring System. We analyzed data obtained from 40 outpatients with diabetes (19 males; age 65 ± 8 years; BMI at 30 ± 5; with a mean HbA1c level at 7.33%), who contributed a mean of 130.6 mg/dL blood glucose level through CGM (BG ranging from 40 to 400 mg/dL). Individuals were eligible for inclusion if they were adults with a diagnosis of T2D patients using CGM. We present the blood glucose history of four selected patients in Supplementary Fig. 2. Ten patients (25% of the participants) were treated with insulin while 27 (67.5% of the participants) were receiving oral or (non-insulin) injectable antidiabetic drugs. The rest of the patients (3 patients, 7.5% of the participants) were treated without oral nor insulin medications. We identified all level 1 hypoglycemic (BG level <80 mg/dL) and hyperglycemic (BG level >180 mg/dL) episodes from the CGM recordings. To facilitate the network training, the BG levels were scaled by 0.01^67^, and we applied a smoothing step on the BG measurements to remove any large spikes that may be caused by patient movement, as suggested in ref. ^68^. An overview of the dataset used in this work can be found in Table 2.

### Predictors and outcome

The primary outcome of interest in this study is the BG values in the future, e.g., 5 min to 1 hr later. We take the BG measured in 30 min (7 BG values) as one input data segment and predict the future BG level after a prediction horizon, a time period from the most recent CGM measurement in the input BG values, as shown in Fig. 1.

### Neural network architectures

We developed new deep-learning methods for patient-specific blood glucose level prediction. We considered three different neural network architectures, including recurrent neural networks (RNNs)^44,69^, gated convolutional neural networks (CNNs)^45^, and self-attention networks (SAN)^46^, as well as three different transfer-learning strategies. We also implemented Gaussian process regression (GP), fully connected feedforward neural networks (FNNs), and support vector machine (SVM) as the baseline models. We implement GP and SVM with the sklearn library^70^. For GP, we use a combined kernel consisting of a constant kernel, a radial basis function kernel and a white noise kernel. For SVM, we use the default hyperparameters. For FNN, we use a 10-neuron-width, 3-layer-depth network. The detailed hyperparameters of the baseline models are optimized via grid search and can be found in the released code repository. To tackle the important issue of data imbalance, we tested four different data augmentation methods, i.e., oversampling by repeating, infusing Gaussian noises, TimeGAN and mixup, on the minority class.

**Fig. 8:**
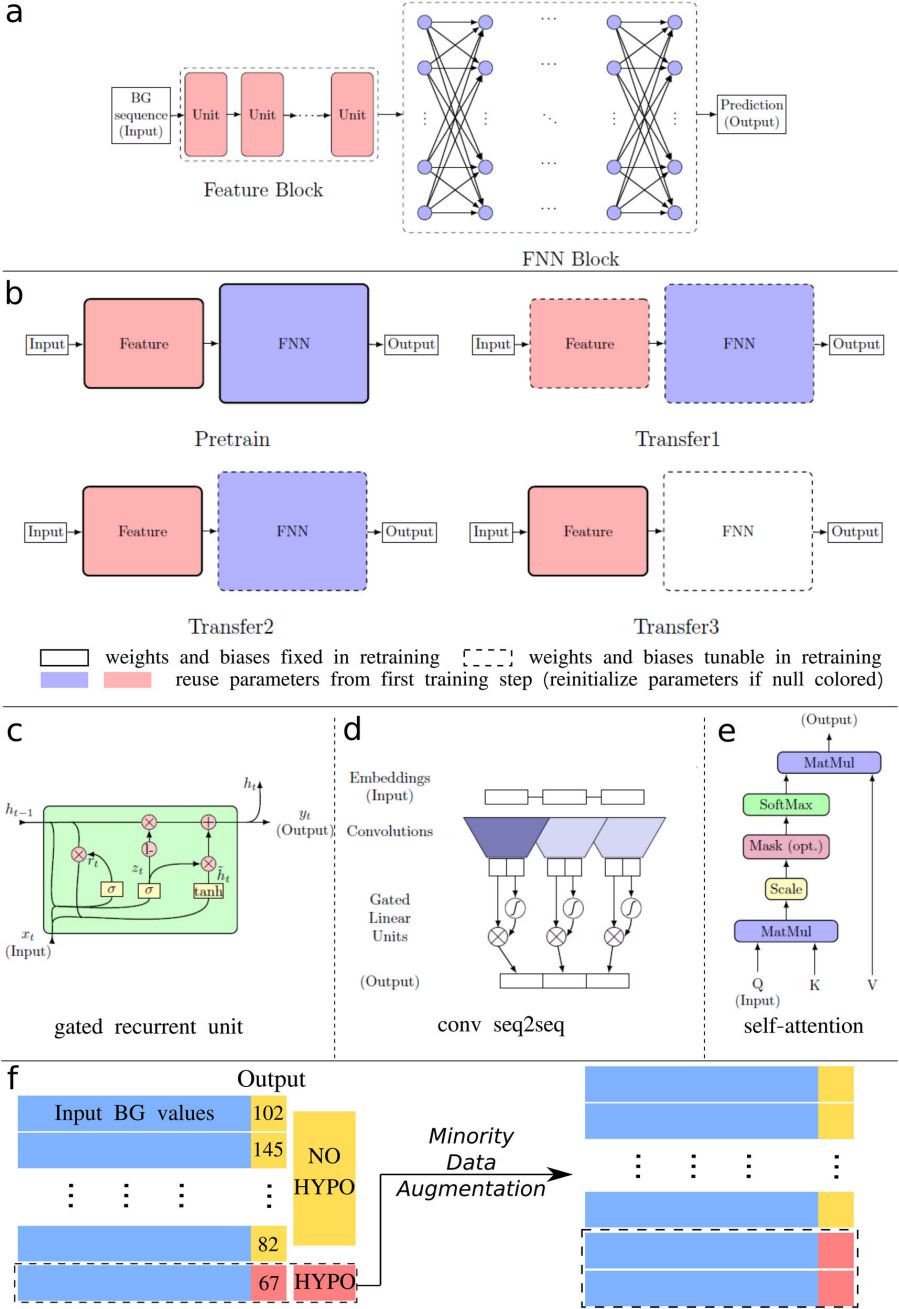
Neural network architectures, transfer-learning methods, and data preprocessing. **a** The general structure of the neural networks implemented, consisting of a feature block and a FNN block. The unit in the feature block is model-specific, for example, when referring to the RNN model, the units are GRUs. **b** Transfer-learning methods implemented, where colored blocks represent reusing neural network parameters (weights and biases) inherited from the pre-training step, the null-colored block in Transfer3 represents re-initializing the network parameters of the FNN block. Blocks bounded with solid lines represent those blocks, of which the network parameters are frozen during retraining step. **c**–**e** Representations of each unit used in different types of neural networks. **c** A gated recurrent unit (GRU) used in the RNN models. **d** A convolutional sequence to sequence (conv seq2seq) unit used in the CNN models. **e** A self-attention unit used in the SAN models. More details of transfer learning can be found in Table 3. **f** Data pre-processing methods employed to deal with data imbalance, the minority data (sequences with hypoglycemia labels) are oversampled to increase model sensitivity for hypoglycemia detection. Output BG values greater then 80 mg/dL are labeled with “no hypoglycemia” (NO HYPO) and those smaller than 80 mg/dL are labeled with “hypoglycemia” (HYPO).

The dominant deep learning method used for sequence learning is the RNN, which is a class of neural networks that allow previous outputs to be used as the inputs of the current step. The cell units in RNNs are usually chosen as long short-term memory units (LSTMs)^69^ and gated recurrent units (GRUs)^44^, which deal with the vanishing gradient problem encountered by traditional RNNs. In addition to RNNs, CNNs and self-attention networks were proposed recently for time series forecasting, and achieved better performance than RNNs for certain tasks. In the gated CNNs, one-dimensional (1-D) convolutional kernels create hierarchical representations over the input time series, in which nearby BG measurements interact at lower layers while distant BG measurements interact at higher layers. The mechanism of attention was first proposed in ref. ^71^ for machine translation, and it has been shown that the network architecture based solely on self-attention mechanism can also be used successfully to compute a representation of the sequence^46^. Self-attention is an attention mechanism to compute a representation of the sequence by relating different positions of a sequence. In the RNNs, the input sequence is fed into the network sequentially, while in CNNs and self-attention networks, the input sequence is fed into the network simultaneously, and thus an embedding of the position of input elements is required^45^. For the hyperparameters in the networks, e.g., the depth and width, we perform a grid search to obtain an optimal set of hyperparameters, see Table 3 for more details. The details of the network architectures used in this study are shown in Fig. 8.

**Table 2:** Baseline characteristics of the study participants and an overview of the blood glucose data. Normally distributed variables are presented in mean ± standard deviation form, otherwise as median (first quartile, third quartile) and mean ± standard deviation form.

Demographics	*N* = 40
Age, years	64.5 (58.8, 70.0), 65.1 ± 8.8
Female, no. (%)	21 (52.5)
Body compositions	
Body mass, kg	81.0 (71.3, 94.2), 84.1 ± 18.7
Height, m	1.64 (1.59, 1.73), 1.66 ± 0.10
BMI, kg/m^2^	29.7 (26.6, 33.1), 30.1 ± 5.1
Hormone levels	
Cortisol, μg/dL	15.9 (13.0, 20.2), 16.1 ± 6.0
Leptin, ng/dL	19.8 (9.57, 31.1), 23.0 ± 17.8
Fasting glucose, mg/dL	117.5 ± 17.9
Insulin, μIU/mL	13.33 ± 13.29
HOMA1-IR	3.51 ± 3.47
Blood glucose data brief	
Data reading length (h)	90 (82, 170), 117 ± 63
Model input BG length (min)	30
Hypoglycemia threshold (mg/dL)	80
Hyperglycemia threshold (mg/dL)	180
HbA1c (%)	7.33 ± 1.31

HOMA1-IR the homeostatic model assessment index for insulin resistance. We choose 80 mg/dL as the hypoglycemia threshold, because recent results by Farrell et al.^57^ have revealed a measurement artifact, i.e., that the real-time CGM underestimates the degree of hypoglycemia by a difference of 10 mg/dL, as shown in Supplementary Fig. 1.

*N*, the number of participants.

**Table 3:** Details of the neural network architectures and transfer-learning models.

	Models	Details
Network architecture	RNN	GRU size 10, 2 GRUs; FNN width 10, 1 FNN layer
	SAN	8 self-attention units; FNN width 10, 4 FNN layers
	CNN	1-D convolutional kernel size 4, 4 conv seq2seq units; FNN width 10, 3 FNN layers
Transfer-learning method	Transfer1	Reuse weights of feature block and FNN block, retrain both blocks
	Transfer2	Reuse weights of feature block and FNN block, retrain FNN block
	Transfer3	Reuse weights of feature block, reinitialize FNN block, retrain FNN block

*GRU* gated recurrent unit, FNN fully connected neural networks, RNN recurrent neural networks, SAN self-attention networks, CNN convolutional neural networks, *conv seq2seq* convolutional sequence to sequence.

### Transfer learning

To address the difficulty of obtaining a sufficient large dataset for each patient, we implemented transfer learning^33–36^ on the three aforementioned neural network architectures. In transfer learning, the training procedure of neural networks includes two steps: first, we pre-train the networks on other patients’ data by excluding the data from the target patient, and then we further fine-tune the network on one part of the target patient’s data, i.e., re-train the network on the training data of the target patient’s blood glucose history. Finally, we test the network on the rest of the data from the target patient. Two commonly used further-training approaches are based on initialization and feature extraction^72^. In the initialization approach, the entire network is trained, while in the feature extraction approach the last few fully connected layers are trained from a random initialization while other layers remain unchanged. In this study, in addition to these two approaches, we consider a third approach by combining these two approaches, i.e., the last few fully connected layers are further trained while other layers remain unchanged. The details of the four transfer learning methods can be found in Fig. 8 and Table 3.

### Imbalanced data

Imbalanced data has been an ubiquitous issue in many fields, causing most methods to yield erroneous predictions strongly biasing towards the majority class. To reduce the hazardous effect of imbalanced data, we can improve the method with various techniques: (i) modifying the imbalanced data set by some mechanisms such as oversampling or undersampling or both to provide a balanced distribution; (ii) designing problem-specific cost matrices to describe the costs for misclassifying any particular data example; (iii) using boosting methods^73,74^. Here, we tested several methods for data augmentation on the training data of the minority class only, i.e., oversampling by repeating, adding Gaussian white noises to the input data, generating synthetic minority samples using TimeGAN^49^ and mixup^48^, respectively. We compared the performance of these preprocessing techniques in terms of four classification metrics, i.e., sensitivity, positive predictive value, specificity and negative predictive value.

### Model validation

For model validation, if the networks are trained on multiple patients, then we used a Leave-one-out cross-validation (LOOCV), i.e., we randomly selected the dataset of one patient to be the test dataset and used the dataset of the remaining patients to train the model. The outcome variables indicate whether or not hypoglycemia or hyperglycemia occurred. The model performance is measured in terms of the prediction accuracy, which is defined as follows,

2 $$\,\text{Accuracy}\,=\frac{{\mathrm{TP}}+{\mathrm{FN}}}{{\mathrm{TP}}+{\mathrm{FN}}+{\mathrm{TN}}+{\mathrm{FP}}},$$

and the area under the receiver operating characteristic curve (AUROC). To calibrate the data augmentation effect on the imbalanced dataset, we computed four classification metrics, sensitivity, positive predictive value (PPV), specificity and negative predictive value (NPV) from the following formulas:

3 $$\begin{array}{ll}\,\text{Sensitivity}\,&=\frac{{\mathrm{TP}}}{{\mathrm{TP}}+{\mathrm{FN}}},\ {\mathrm{PPV}}=\frac{{\mathrm{TP}}}{{\mathrm{TP}}+{\mathrm{FP}}},\\ \,\text{Specificity}\,&=\frac{{\mathrm{TN}}}{{\mathrm{TN}}+{\mathrm{FP}}},\ {\mathrm{NPV}}=\frac{{\mathrm{TN}}}{{\mathrm{TN}}+{\mathrm{FN}}}.\end{array}$$

where TP denotes the number of true positives, FP denotes that of false positives, TN denotes the number of true negatives, and FN denotes that of false negatives.

### Reporting summary

Further information on research design is available in the Nature Research Reporting Summary linked to this article.

## Supplementary information

Supplemental Material

Reporting Summary

## Data Availability

The dataset used in the current study provided by Beth Israel Deaconess Medical Center (BIDMC) is not publicly available, due to reasonable privacy and security concerns. The data is not easily redistributable to researchers other than those engaged in the Institutional Review Board-approved research collaborations with Beth Israel Deaconess Medical Center (BIDMC).

## Code availability

The code is available on a GitHub repository: https://github.com/yixiangD/AccurateBG.
