# Supplementary material for: Deep transfer learning and data augmentation improve glucose levels prediction in type 2 diabetes patients
Source: NPJ Digit Med. 2021 Jul 14;4:109. doi: 10.1038/s41746-021-00480-x (PMC8280162; doi:10.1038/s41746-021-00480-x)
Supplement: Supplementary file 1 — Supplemental Material [file 41746_2021_480_MOESM1_ESM.pdf]

Supplementary Material: Deep transfer learning and data augmentation improve glucose levels prediction in type 2 diabetes patients

Yixiang Deng, Lu Lu, Laura Aponte, Angeliki M. Angelidi, Vera Novak, George Em Karniadakis, Christos S. Mantzoros

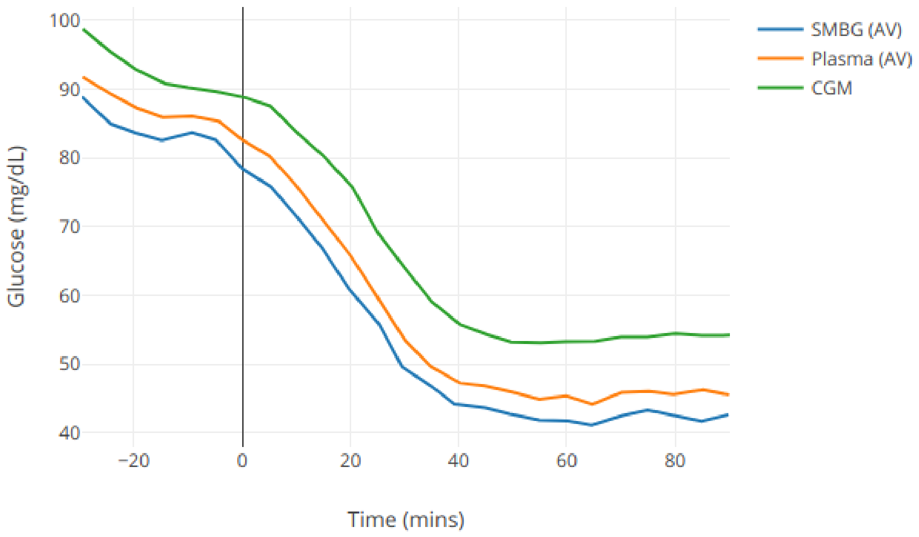

Supplementary Figure 1: **Glucose data (mean) during hyperinsulinemic hypoglycemic clamps**. Orange curve represents AV plasma glucose; blue curve, AV blood tested via SMBG; green curve, CGM. AV, arterialized venous. SMBG, self-monitoring of blood glucose. Figure reproduced from Farrell et al. (57).

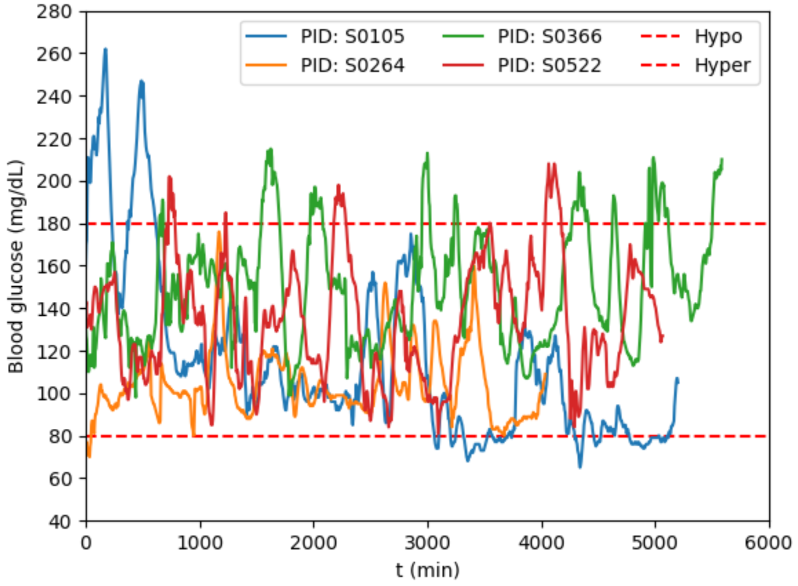

Supplementary Figure 2: **Blood glucose (BG) history measured by CGM of four selected patients**. Each colored solid line represents the blood glucose history of one patient, the ID of which is marked in the figure caption. The red dash lines denote the critical threshold of hypoglycemia (BG = 80 mg/dL) and hyperglycemia (BG = 180 mg/dL), respectively. PID, patient's ID.

# Training Data from Target Patient	Logistic Regression	GP	SVM	FNN	Our Best
50	0.990±0.003	0.988±0.003	0.990±0.003	0.990±0.003	0.988±0.003
100	0.990±0.003	0.989±0.003	0.990±0.003	0.991±0.002	0.990±0.003
400	0.992±0.003	0.991±0.003	0.991±0.003	0.992±0.002	0.992±0.002
1000	0.973±0.015	0.971±0.016	0.973±0.015	0.973±0.016	0.993±0.002

Supplementary Table 1: **Hypoglycemia detection (binary classification,** hypoglycemia vs. no hypoglycemia**) accuracy** (mean ± standard error) **of different methods with different numbers of training data from the target patient, for prediction horizon at 30 minutes and training models on the cohort** (patients participated in this study) **data.** GP, Gaussian Process; SVM, supporting vector machine; FNN, the best fully-connected neural networks; Our Best, the best model among the combinations of three proposed architectures (RNN, CNN and SAN) and four transfer learning methods (Pretrain, Transfer1, Transfer2 and Transfer3). Details of the network architectures and transfer learning methods can be found in Fig. 8 and Table 2.

# Training Data from Target Patient	Logistic Regression	GPC	SVC	FNN	Our Best
50	0.924±0.007	0.919±0.007	0.924±0.006	0.926±0.006	0.929±0.006
100	0.924±0.007	0.919±0.008	0.924±0.007	0.927±0.007	0.926±0.007
400	0.929±0.007	0.926±0.007	0.927±0.007	0.931±0.006	0.930±0.007
1000	0.939±0.011	0.938±0.012	0.938±0.011	0.941±0.011	0.948±0.010

Supplementary Table 2: **Hypoglycemia and hyperglycemia detection (three-class classification) accuracy** (mean ± standard error) **of different methods with different numbers of training data from the target patient, for prediction horizon at 30 minutes and training models on the cohort** (patients participated in this study) **data.** In *three-class classification*, the output label of prediction is one out of three labels, "hypoglycemia", "normoglycemia" and "hyperglycemia", at any given time. GP, Gaussian Process; SVM, supporting vector machine; FNN, the best fully-connected neural networks. Our Best, the best model among the combinations of three proposed architectures (RNN, CNN and SAN) and four transfer learning methods (Pretrain, Transfer1, Transfer2 and Transfer3). Details of the network architectures and transfer learning methods can be found in Fig. 8 and Table 2.

# Training Data from Target Patient	Logistic Regression	GP	SVM	FNN	Our Best
50	0.990±0.003	0.988±0.003	0.990±0.003	0.990±0.002	0.988±0.003
100	0.990±0.003	0.988±0.003	0.990±0.003	0.990±0.003	0.990±0.003
400	0.992±0.003	0.992±0.003	0.991±0.003	0.992±0.002	0.992±0.002
1000	0.988±0.006	0.988±0.006	0.988±0.006	0.988±0.006	0.992±0.003

Supplementary Table 3: **Hypoglycemia detection (binary classification) accuracy** (mean ± standard error) **of different methods with different numbers of training data from the target patient, for prediction horizon at *60 minutes*.** GP, Gaussian Process; SVM, supporting vector machine; FNN, the best fully-connected neural networks; Our Best, the best model among the combinations of three proposed architectures (RNN, CNN and SAN) and four transfer learning methods (Pretrain, Transfer1, Transfer2 and Transfer3). Details of the network architectures and transfer learning methods can be found in Fig. 8 and Table 2.

# Training Data from Target Patient	Logistic Regression	GPC	SVC	FNN	Our Best
50	0.924±0.007	0.918±0.007	0.924±0.006	0.927±0.006	0.929±0.006
100	0.924±0.007	0.919±0.007	0.924±0.007	0.925±0.007	0.926±0.007
400	0.929±0.007	0.926±0.007	0.927±0.007	0.929±0.006	0.930±0.007
1000	0.939±0.011	0.940±0.010	0.938±0.011	0.940±0.010	0.944±0.010

Supplementary Table 4: **Hypoglycemia and hyperglycemia detection (three-class classification) accuracy** (mean ± standard error) **of different methods with different numbers of training data from the target patient, for prediction horizon at *60 minutes*.** GP, Gaussian Process; SVM, supporting vector machine; FNN, the best fully-connected neural networks; Our Best, the best model among the combinations of three proposed architectures (RNN, CNN and SAN) and four transfer learning methods (Pretrain, Transfer1, Transfer2 and Transfer3). Details of the network architectures and transfer learning methods can be found in Fig. 8 and Table 2.

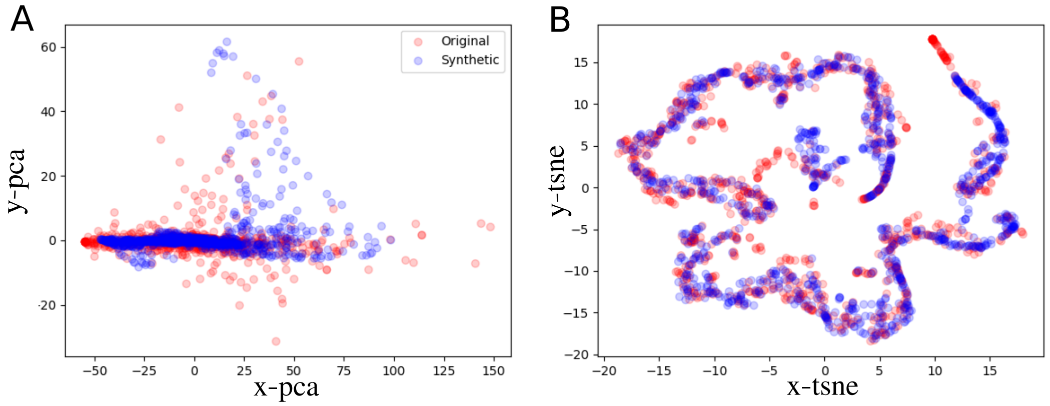

Supplementary Figure 3: **The visualization results of the hypoglycemia samples generated by TimeGAN**. (**A**) PCA (principle component analysis) and (**B**) T-SNE. The discriminative score output from our trained TimeGAN is around 0.10.

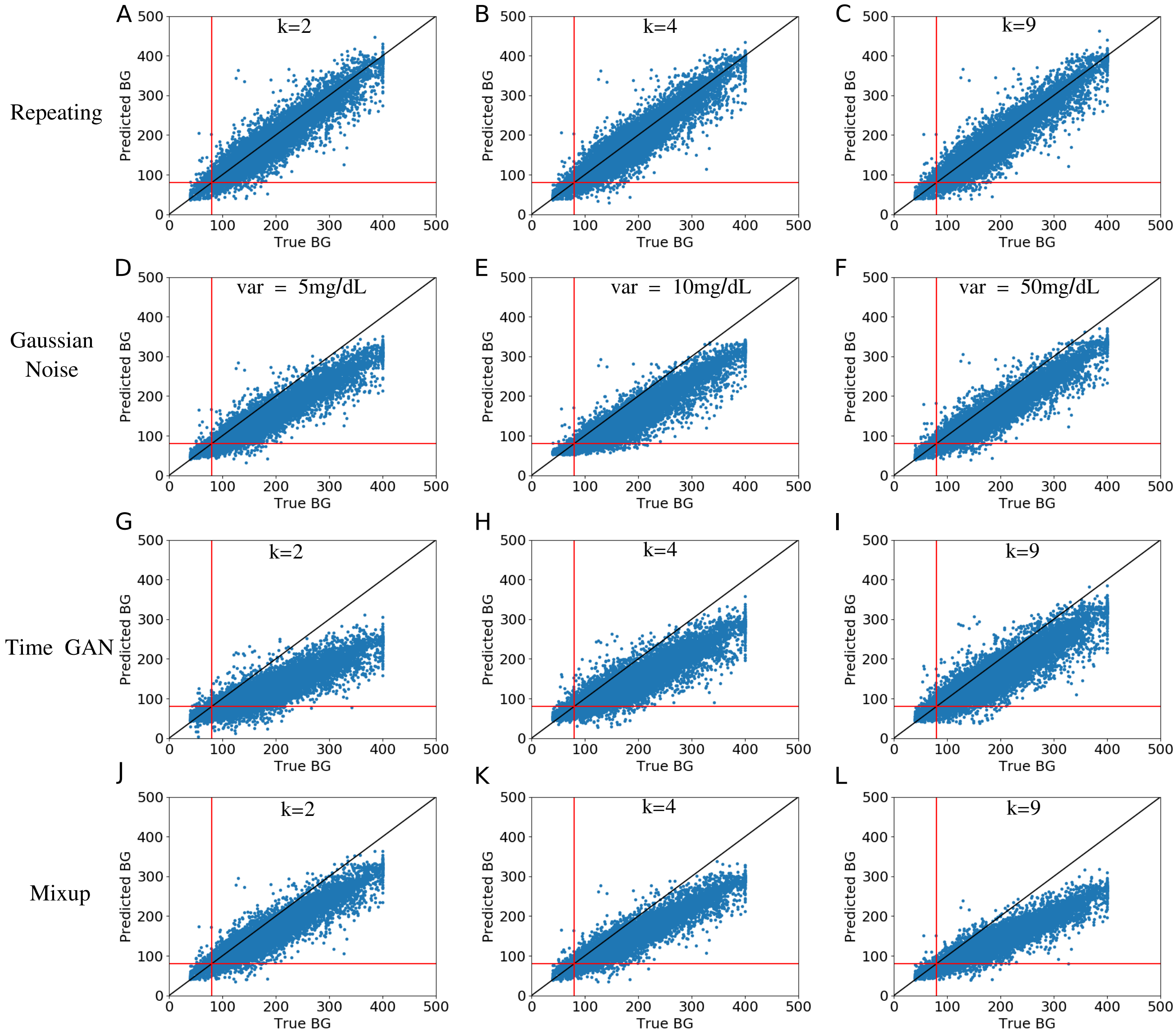

Supplementary Figure 4: **True BG measured by CGM vs. BG prediction from our model trained with data augmentation on the minority training data, given different data augmentation/preprocessing methods**. Minority training data is augmented with (**A** to **C**) oversampling by repeating, (**D** to **F**) Gaussian white noise infusion to a copy of minority training data, (**G** to **I**) TimeGAN (49) and (**J** to **L**) mixup (48). Minority training data size is (A, G, J) doubled or (B, E, K) becomes 4 times or (C, I, L) becomes 9 times of its original size after data augmentation. Minority training data size is doubled in (D to F), but different noise level is considered in each figure, i.e., the new copy of minority data is generated by infusing Gaussian white noise with (D) variance 5mg/dL, (E) variance 10 mg/dL and (F) variance 50 mg/dL to the original minority training data. We note that with data augmentation methods, such as Gaussian noise, TimeGAN and mixup, which introduce synthetic data, the model sensitivity increases to more than 90%. Specifically, for TimeGAN and mixup, increasing the minority data fold k, further increases the model sensitivity. However, the increase in sensitivity sacrifices the precision of the minority class (hypoglycemia), as indicated by the inclination of the scattering points, i.e., the (True BG, Predicted BG) pairs, towards x axis shown in the figure.

Supplementary Table 5: **Comparison of four classification scores using four different data augmentation methods on minority class**. Classification scores for model trained on dataset with (**A**) repeating, (**B**) Gaussian noise, (**C**) TimeGAN (**D**) mixup ($\alpha = 2$). PPV, positive predictive value. "raw", model without data augmentation. NPV, negative predictive value. Noise Var, noise variance. Positive class is the class of samples labeled with hypoglycemia, which is also the minority class.

(**A**): Repeating

Fold	Sensitivity	PPV	Specificity	NPV
raw	$0.7688 \pm 1.12 \times 10^{-2}$	$0.7466 \pm 5.50 \times 10^{-3}$	$0.9917 \pm 4.00 \times 10^{-4}$	$0.9906 \pm 4.00 \times 10^{-4}$
2	$0.7907 \pm 7.10 \times 10^{-3}$	$0.7309 \pm 7.10 \times 10^{-3}$	$0.9925 \pm 3.00 \times 10^{-4}$	$0.9895 \pm 5.00 \times 10^{-4}$
4	$0.8123 \pm 1.26 \times 10^{-2}$	$0.7038 \pm 8.80 \times 10^{-3}$	$0.9932 \pm 4.00 \times 10^{-4}$	$0.9877 \pm 7.00 \times 10^{-4}$
9	$0.8443 \pm 7.40 \times 10^{-3}$	$0.6583 \pm 8.60 \times 10^{-3}$	$0.9944 \pm 3.00 \times 10^{-4}$	$0.9843 \pm 7.00 \times 10^{-4}$

(**B**): Gaussian Noise

Noise var	Sensitivity	PPV	Specificity	NPV
raw	$0.7688 \pm 1.12 \times 10^{-2}$	$0.7466 \pm 5.50 \times 10^{-3}$	$0.9917 \pm 4.00 \times 10^{-4}$	$0.9906 \pm 4.00 \times 10^{-4}$
5	$0.8878 \pm 2.22 \times 10^{-2}$	$0.5539 \pm 5.85 \times 10^{-2}$	$0.9959 \pm 8.00 \times 10^{-4}$	$0.9735 \pm 6.80 \times 10^{-3}$
10	$0.9312 \pm 1.75 \times 10^{-2}$	$0.3859 \pm 6.53 \times 10^{-2}$	$0.9974 \pm 6.00 \times 10^{-4}$	$0.9443 \pm 1.41 \times 10^{-2}$
50	$0.9679 \pm 1.26 \times 10^{-2}$	$0.2688 \pm 3.95 \times 10^{-2}$	$0.9987 \pm 5.00 \times 10^{-4}$	$0.9022 \pm 2.18 \times 10^{-2}$

(**C**): TimeGAN

Fold	Sensitivity	PPV	Specificity	NPV
raw	$0.7688 \pm 1.12 \times 10^{-2}$	$0.7466 \pm 5.50 \times 10^{-3}$	$0.9917 \pm 4.00 \times 10^{-4}$	$0.9906 \pm 4.00 \times 10^{-4}$
2	$0.8947 \pm 8.90 \times 10^{-3}$	$0.5433 \pm 2.34 \times 10^{-2}$	$0.9961 \pm 3.00 \times 10^{-4}$	$0.9729 \pm 2.80 \times 10^{-3}$
4	$0.9386 \pm 3.60 \times 10^{-3}$	$0.3061 \pm 1.57 \times 10^{-2}$	$0.9976 \pm 1.00 \times 10^{-4}$	$0.9233 \pm 5.70 \times 10^{-3}$
9	$0.8976 \pm 2.49 \times 10^{-2}$	$0.2096 \pm 1.89 \times 10^{-2}$	$0.9958 \pm 9.00 \times 10^{-4}$	$0.8769 \pm 1.72 \times 10^{-2}$

(**D**): mixup, $\alpha = 2$

Fold	Sensitivity	PPV	Specificity	NPV
raw	$0.7688 \pm 1.12 \times 10^{-2}$	$0.7466 \pm 5.50 \times 10^{-3}$	$0.9917 \pm 4.00 \times 10^{-4}$	$0.9906 \pm 4.00 \times 10^{-4}$
2	$0.9068 \pm 4.74 \times 10^{-2}$	$0.4987 \pm 1.07 \times 10^{-1}$	$0.9966 \pm 1.70 \times 10^{-3}$	$0.9639 \pm 1.40 \times 10^{-2}$
4	$0.9339 \pm 3.65 \times 10^{-2}$	$0.4181 \pm 1.22 \times 10^{-1}$	$0.9975 \pm 1.30 \times 10^{-3}$	$0.9459 \pm 2.46 \times 10^{-2}$
9	$0.9366 \pm 1.94 \times 10^{-2}$	$0.3905 \pm 6.10 \times 10^{-2}$	$0.9976 \pm 7.00 \times 10^{-4}$	$0.9454 \pm 1.32 \times 10^{-2}$

Supplementary Table 6: **Comparison of three classification scores for minority class between two mixup models and the original dataset**. Classification scores for model trained on dataset with (**A**) no data augmentation, (**B**) minority data augmentation by mixup (α = 0.4) and (**C**) minority data augmentation by mixup (α = 2). PPV, positive predictive value. NPV, negative predictive value. Positive class is the class of samples labeled with hypoglycemia, which is also the minority class.

(**A**): No data augmentation

Prediction horizon (min)	Sensitivity	PPV	Specificity	NPV	Accuracy
5	0.9437±2.40e-3	0.8972±2.40e-3	0.9961±1.00e-4	0.9980±1.00e-4	0.9943±1.00e-4
15	0.8236±2.70e-3	0.7855±4.00e-3	0.9919±2.00e-4	0.9937±1.00e-4	0.9861±2.00e-4
30	0.6777±8.60e-3	0.6843±5.60e-3	0.9888±4.00e-4	0.9884±3.00e-4	0.9780±2.00e-4
45	0.5269±1.21e-2	0.6126±1.02e-2	0.9881±6.00e-4	0.9832±4.00e-4	0.9721±5.00e-4
60	0.3942±1.84e-2	0.5423±6.00e-3	0.9881±4.00e-4	0.9786±6.00e-4	0.9676±4.00e-4

(**B**): Minority data augmentation by mixup: α = 0.4

Prediction horizon (min)	Sensitivity	PPV	Specificity	NPV	Accuracy
5	0.9633±1.08e-2	0.8455±4.38e-2	0.9935±2.30e-3	0.9987±4.00e-4	0.9925±1.80e-3
15	0.9121±5.75e-2	0.5767±1.56e-1	0.9704±1.85e-2	0.9968±2.00e-3	0.9684±1.60e-2
30	0.8996±3.27e-2	0.3576±6.91e-2	0.9378±2.15e-2	0.9962±1.20e-3	0.9365±1.97e-2
45	0.8911±3.51e-2	0.2234±5.67e-2	0.8763±5.05e-2	0.9956±1.20e-3	0.8768±4.76e-2
60	0.9086±5.10e-3	0.1342±5.00e-3	0.7902±9.20e-3	0.9959±2.00e-4	0.7942±8.80e-3

(**C**): Minority data augmentation by mixup: α = 2

Prediction horizon (min)	Sensitivity	PPV	Specificity	NPV	Accuracy
5	0.9447±3.05e-2	0.8794±3.53e-2	0.9952±1.70e-3	0.9980±1.10e-3	0.9935±7.00e-4
15	0.9056±3.33e-2	0.6341±8.00e-2	0.9801±7.50e-3	0.9966±1.20e-3	0.9776±6.10e-3
30	0.8695±2.55e-2	0.4216±4.92e-2	0.9559±1.05e-2	0.9951±9.00e-4	0.9529±9.30e-3
45	0.8808±3.00e-2	0.2478±5.17e-2	0.8982±2.87e-2	0.9953±1.00e-3	0.8976±2.68e-2
60	0.9053±2.74e-2	0.1363±2.76e-2	0.7828±5.99e-2	0.9958±9.00e-4	0.7871±5.70e-2
