# Supplementary material for: Deep transfer learning and data augmentation improve glucose levels prediction in type 2 diabetes patients
Source: NPJ Digit Med. 2021 Jul 14;4:109. doi: 10.1038/s41746-021-00480-x (PMC8280162; doi:10.1038/s41746-021-00480-x)
Supplement: Supplementary file 2 — Reporting Summary [file 41746_2021_480_MOESM2_ESM.pdf]

nature research

Corresponding author(s): George Em Karniadakis, Christos S Mantzoros

Last updated by author(s): May 6, 2021

Reporting Summary

Nature Research wishes to improve the reproducibility of the work that we publish. This form provides structure for consistency and transparency in reporting. For further information on Nature Research policies, see our Editorial Policies and the Editorial Policy Checklist.

Statistics

For all statistical analyses, confirm that the following items are present in the figure legend, table legend, main text, or Methods section.

n/a	Confirmed	
☐	☒	The exact sample size (*n*) for each experimental group/condition, given as a discrete number and unit of measurement
☐	☒	A statement on whether measurements were taken from distinct samples or whether the same sample was measured repeatedly
☐	☒	The statistical test(s) used AND whether they are one- or two-sided<br>*Only common tests should be described solely by name; describe more complex techniques in the Methods section.*
☐	☒	A description of all covariates tested
☐	☒	A description of any assumptions or corrections, such as tests of normality and adjustment for multiple comparisons
☐	☒	A full description of the statistical parameters including central tendency (e.g. means) or other basic estimates (e.g. regression coefficient) AND variation (e.g. standard deviation) or associated estimates of uncertainty (e.g. confidence intervals)
☐	☒	For null hypothesis testing, the test statistic (e.g. *F*, *t*, *r*) with confidence intervals, effect sizes, degrees of freedom and *P* value noted<br>*Give P values as exact values whenever suitable.*
☒	☐	For Bayesian analysis, information on the choice of priors and Markov chain Monte Carlo settings
☒	☐	For hierarchical and complex designs, identification of the appropriate level for tests and full reporting of outcomes
☒	☐	Estimates of effect sizes (e.g. Cohen's *d*, Pearson's *r*), indicating how they were calculated

Our web collection on statistics for biologists contains articles on many of the points above.

Software and code

Policy information about availability of computer code

Data collection	We use CGM sensor brand: Medtronic, Inc. and version: iPro 2 for the data collection.
Data analysis	We used a custom algorithm, which is made publicly available on GitHub and the link is stated in the "Code Availability" section.

For manuscripts utilizing custom algorithms or software that are central to the research but not yet described in published literature, software must be made available to editors and reviewers. We strongly encourage code deposition in a community repository (e.g. GitHub). See the Nature Research guidelines for submitting code & software for further information.

Data

Policy information about availability of data

All manuscripts must include a data availability statement. This statement should provide the following information, where applicable:

- Accession codes, unique identifiers, or web links for publicly available datasets
- A list of figures that have associated raw data
- A description of any restrictions on data availability

The dataset used in the current study is not publicly available, due to reasonable privacy and security concerns. The data is not easily redistributable to researchers other than those engaged in the Institutional Review Board-approved research collaborations with the named medical institute.

Field-specific reporting

Please select the one below that is the best fit for your research. If you are not sure, read the appropriate sections before making your selection.

☒ Life sciences ☐ Behavioural & social sciences ☐ Ecological, evolutionary & environmental sciences

For a reference copy of the document with all sections, see nature.com/documents/nr-reporting-summary-flat.pdf

Life sciences study design

All studies must disclose on these points even when the disclosure is negative.

Sample size	60 patients are recruited for the study, 40 of them are used in the final result.
Data exclusions	Patients with pre-diabetes condition is excluded.
Replication	Yes, replication is successful.
Randomization	Yes, random allocation is conducted.
Blinding	Yes, the investigators are blind to the participants.

Reporting for specific materials, systems and methods

We require information from authors about some types of materials, experimental systems and methods used in many studies. Here, indicate whether each material, system or method listed is relevant to your study. If you are not sure if a list item applies to your research, read the appropriate section before selecting a response.

Materials & experimental systems

n/a	Involved in the study	
☒	☐	Antibodies
☒	☐	Eukaryotic cell lines
☒	☐	Palaeontology and archaeology
☒	☐	Animals and other organisms
☒	☐	Human research participants
☐	☒	Clinical data
☒	☐	Dual use research of concern

Methods

n/a	Involved in the study	
☒	☐	ChIP-seq
☒	☐	Flow cytometry
☒	☐	MRI-based neuroimaging

Clinical data

Policy information about clinical studies

All manuscripts should comply with the ICMJE guidelines for publication of clinical research and a completed CONSORT checklist must be included with all submissions.

Clinical trial registration	It is not registered on clinical trials.gov, but was approved approved by the institutional review board (IRB) of the Beth Israel Deaconess Medical Center.
Study protocol	Available on Physionet https://physionet.org/content/cded/1.0.0/
Data collection	Dates: 7/1/2008-6/30/2013<br>Site: BIDMC<br>Observational study on cerebromicrovascular disease in elderly with diabetes using CGM data.
Outcomes	To determine the effects of type 2 DM on regional gray and white matter and cerebrospinal fluid volumes. The study in this work used the auxiliary data (CGM) in the main study. The primary outcome is to develop an algorithm that provides accurate blood glucose level estimation within 1 hour in the future using historical blood glucose levels in 30 min. We assess the performance of our algorithm through calculating the errors and accuracy, as well as comparing to existing algorithms for short-term blood glucose level prediction.
